# Acute gastric volvulus following rapid and incomplete chewing of vegetables: A case report

**DOI:** 10.1016/j.radcr.2021.10.003

**Published:** 2021-10-31

**Authors:** Arash Pour Mohammad, Milad Gholizadeh Mesgarha, Mahya Naderkhani, Rasoul Sarmadi, Elham Zarei

**Affiliations:** a Faculty of Medicine, Iran University of Medical Sciences (IUMS), Tehran, Iran; b Emergency Medicine Management Research Center, Iran University of Medical Sciences, Tehran, Iran; c Department of Surgery, Firoozabadi hospital, Iran University of Medical Sciences, Tehran, Iran; d Ali-Asghar Children Hospital, Department of Radiology, School of Medicine, Iran University of Medical Sciences, Tehran, Iran

**Keywords:** Acute abdomen, Gastric volvulus, Mesenteroaxial, Incomplete mastication, Abdominal X_ray

## Abstract

One of the rare but serious causes of acute abdomen is gastric volvulus. It is considered an emergent surgical condition when it takes place acutely due to the risk of gastric strangulation, gangrene, and perforation. In this study, we introduce a case of a previously healthy young adult patient who presented with sudden severe epigastric and left upper quadrant abdominal pain along with nausea and retching following insufficient mastication and rapid swallowing of large amounts of vegetables. Radiological studies with chest and abdominal X-rays were in favor of acute gastric outlet obstruction and finally, laparotomy confirmed the diagnosis of acute, primary mesenteroaxial gastric volvulus. We postulated a probable justifying mechanism of the presence of a flaccid gastrocolic ligament (found through the laparotomy) besides rapid entrance of great pieces of vegetables into the stomach precipitated instant gastric rotation.

## Introduction

Gastric volvulus is an uncommon but potentially life-threatening clinical condition, defined as a pathological rotation of the stomach along its long (organoaxial) or short (mesenteroaxial) axis leading to the gastric inlet and outlet obstruction at variable levels [1,2]. It is regarded as a surgical emergency when it occurs in an acute setting, causing obstruction, ischemia (strangulation) then necrosis, and eventually perforation with stomach rotation of more than 180 degrees [2,3]. Based on the existence of underlying causes, gastric volvulus is classified as primary (30%) and secondary (70%); the primary one is considered when there is no defect in the diaphragm or pathology in adjacent organs but probably it is attributed to weakness or laxity of gastric supports (gastrocolic, gastrosplenic, gastrophrenic and gastrohepatic ligaments), and the secondary one occurs while there are structural or functional gastric disorders or defects in the adjacent anatomies including diaphragm and spleen, which among them, the most common association is with paraesophageal hernia [1,3]. Herein, we provide an atypical presentation of an acute primary mesenteroaxial volvulus in a young adult patient and then discuss its possible pathophysiological mechanism with a brief review of the literature.

## Case report

**Fig. 1:**
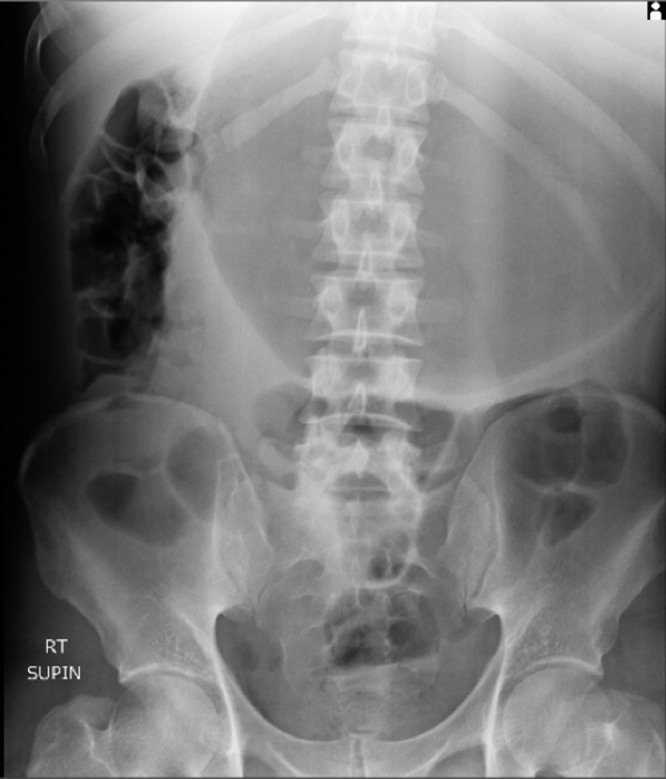
Abdominal X-ray (supine) shows a massively dilated stomach in an expected intraabdominal location with collapsed small bowel loops.

**Fig. 2:**
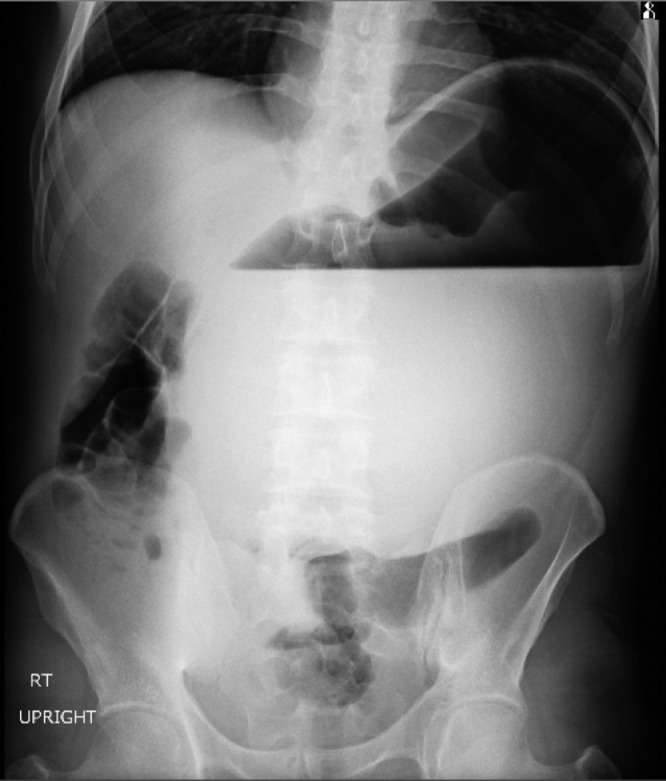
Abdominal X-ray (upright) demonstrates abnormal position of gastric cardia and double bubble gastric air-fluid levels.

**Fig. 3:**
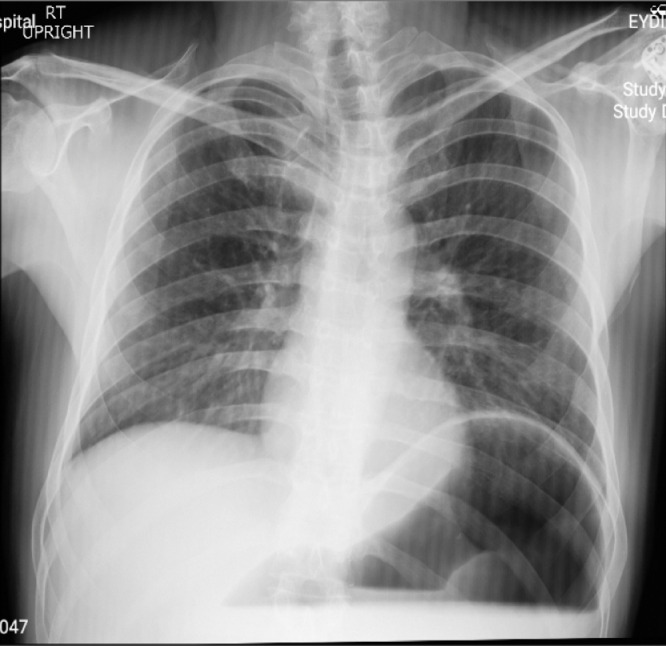
Upright chest X-ray indicates no evidence of pneumoperitoneum but the distended stomach is obvious along with an elevation of the left hemi-diaphragm.

**Fig. 4:**
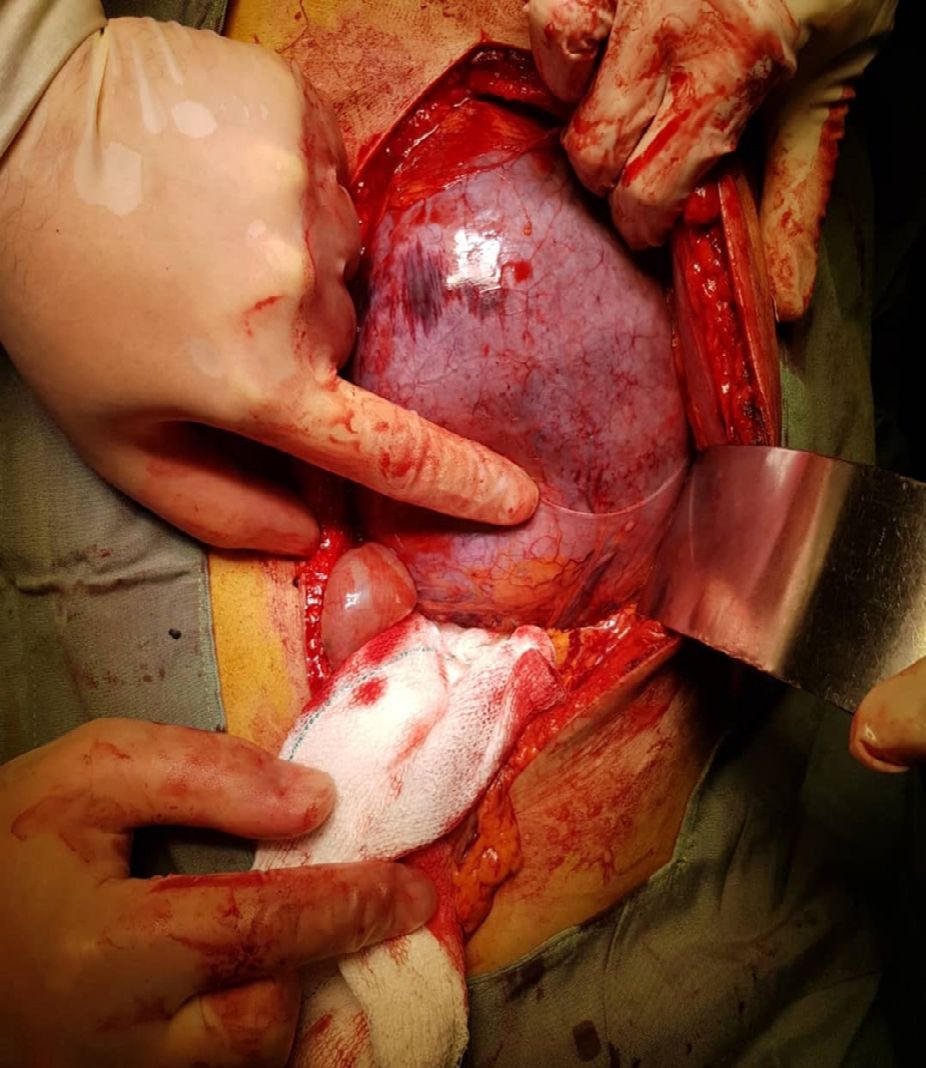
A picture of the stomach during laparotomy was shown and partial gastrectomy was performed due to evidence of gastric necrosis.

A 32-year-old man presented to the emergency department of the hospital with the complaint of severe acute abdominal pain. The patient felt completely healthy until the afternoon of the day before the presentation when he was eating two plates of uncooked vegetables such as parsley, basil, and coriander; that were ingested without enough mastication, and after about ten minutes, he developed sudden onset, severe abdominal pain located at the epigastric and left upper quadrant (LUQ) region. Then, about ten minutes later he noticed an abdominal distention feeling a mass at the LUQ area and acute dyspnea ensued. His abdominal pain was sharp in quality and he rated the intensity of the pain at 10 of 10 (on a scale from 1 to 10, with 0 representing “no pain” and 10 “very much pain”), but no radiation. It was partially alleviated by the knee-chest position and was exacerbated by the supine position. He also reported having nausea and retching but no emesis. The patient's medical history was mild dyspepsia from 20 years ago which was under treatment by Ranitidine consumed as needed and surgical history of appendectomy 4 years ago. There was no significant and relevant family, allergic, social, or habitual history. Upon physical examination; the patient was terrifically ill and was crying and urging for help desperately while he had a knee-chest position on the ground. In vital signs, he had a blood pressure of 110/85 mmHg, pulse rate of 96 beats per minute, a temperature of 36.5 centigrade, and respiratory rate of 28 breaths per minute. On abdominal examination, upon inspection, there was a local distention at the LUQ and epigastric region. The patient refused deep palpation and had a voluntary guarding abdomen but on light palpation, a severely tender mass was found at the LUQ. Other systemic examinations were unremarkable. In laboratory data, the white-cell count was 8400 per cubic millimeter, with 77.2 % neutrophils, 16.4 % lymphocytes. The hemoglobin level was 15 g per deciliter, and the platelet count was 169,000 per cubic millimeter. The serum level of sodium was 139 mmol per liter, potassium level 4.7 mmol per liter, Blood urea nitrogen level 23 mg per deciliter, creatinine level 1 mg per deciliter, and blood glucose 111 milligrams per deciliter. Venous blood gas analysis showed a pH of 7.33, partial pressure of carbon dioxide of 43.9 mm Hg, and a bicarbonate level of 23.1 mmol per liter. The liver function test, amylase, lipase, erythrocyte sedimentation rate, C-reactive protein, prothrombin time, partial thromboplastin time, and the international normalized ratio were within the normal range. Abdominal X-ray (supine and upright) showed a massively dilated stomach in an expected intraabdominal location with collapsed small bowel loops (Fig. 1 and 2). Abnormal position of gastric cardia and double bubble gastric air-fluid levels were also found (Fig. 2). Upright chest X-ray demonstrated no evidence of pneumoperitoneum but the distended stomach was obvious (Fig. 3). These findings were in favor of gastric outlet obstruction (G.O.O). In spite, there was no classic appearance of complete gastric volvulus, due to clinical history of acute onset of severe epigastric pain, these findings were suggestive of G.O.O due to acute gastric volvulus. According to the aforementioned findings, a nasogastric tube was tried to assist gastric decompression but failed to pass through which was further in favor of gastric volvulus. Ceftriaxone and metronidazole were administered; thereafter, the patient underwent laparotomy (Fig. 4). During the operation, the diagnosis of acute mesenteroaxial volvulus was confirmed and its reduction was done. Subsequently, partial gastrectomy of the gangrenous part and then gastropexy was performed. The postoperative course was uncomplicated and the patient was discharged in generally good condition. At outpatient follow-up after 2 weeks of discharge, he was doing well.

## Discussion

Presented here was a case of a young adult patient who developed classic symptoms of acute gastric volvulus: severe sudden epigastric pain, intractable retching without vomiting, and inability to pass a nasogastric tube (Bouchard's triad) [4]; Nevertheless, given this clinical and then radiological findings, we considered acute gastric outlet obstruction as a primary diagnosis and in approach to that in the context of the patient presentation, acute gastric volvulus and then phytobezoar were presumed as underlying causes. Eventually, laparotomy made the final diagnosis of acute mesenteroaxial volvulus and ruled out phytobezoar. In addition, further laparotomy findings showed elongated and lax gastrocolic ligament but no evidence of other pathologies in the stomach or its adjacent organs which proved that volvulus was from a primary source. As a result, we hypothesized that the presence of a flaccid gastrocolic ligament on the one hand and the sudden entrance of great pieces of vegetables to the stomach which increased gastric motility, on the other hand, paved the way for a sudden rotation of the stomach. To the best of our knowledge, this patient was the first case who had this unusual presentation of gastric volvulus; accordingly, further observation and studies are required to evaluate our hypothesis.

As mentioned in the case presentation, some gangrenous parts of the stomach were noted which led to partial gastrectomy; the fact that underscores the importance of early diagnosis of this surgical emergency. Gastric volvulus rarity and numerous causes of sudden abdominal pain result in a low clinical index of suspicion for this entity [2,3], therefore consideration of this condition in the differential diagnosis of a patient with acute epigastric pain and early employment of radiological modality is crucial. Upright abdominal X-ray may indicate double air-fluid levels in the antrum and fundus, or a single air bubble without further luminal gas and supine abdominal X-ray shows a distended fluid-filled stomach with a scarcity of distal GI gases [1,3]. Chest X-ray can demonstrate hiatus hernia, retrocardiac air bubble, or large air-fluid level in the chest [5,6]. As a result, although findings on plain radiography can be a useful guide for diagnosis of gastric volvulus, they are not highly specific nor sensitive; as was true about our case. According to the literature, abdominal CT scan is currently the modality of choice for the diagnosis of gastric volvulus with high sensitivity and specificity, superior to the barium study, endoscopy, and the other modalities; there are 2 signs in abdominal CT scan of gastric volvulus used in its diagnosis: gastropyloric transition zone and abnormal location of the antrum. Other advantages of CT scan consist of the definition of the anatomical defect which allows surgery planning, assessment of gastric viability, and perforation which aids decision regarding the urgency of operative intervention and its 24 hours accessibility and rapidity [6], [7], [8], [9]. Although CT scan was accessible in our center, we decided to make an emergent transfer of the patient to the operating room regarding his critical condition and considerable suspicion of gastric volvulus based on the presentation and X-rays findings.

Based on the review of the literature, immediate surgical intervention comprising emergency laparotomy is recommended following diagnosis of acute gastric volvulus [2]. The reduction procedure is followed by a wide combination of procedures, such as simple gastropexy, gastropexy with division of gastrocolic omentum, partial gastrectomy, diaphragmatic crura reapproximation and hiatal repair, and fundo-antral gastrogastrostomy (Opolzer's technique) [10]. Assessment of gastric viability within the operation is crucial to assist in surgery planning and resecting the gangrenous portion as was the case in our patient [11]. Conservative approaches like endoscopic reduction are confined to elderly patients with a less physiologic reserve and chronic gastric volvulus [1].

Recently, laparoscopy is gaining more attention in the treatment of gastric volvulus and some studies have demonstrated its satisfactory outcomes and lower downtime as an alternative; however, data comparing laparoscopy to open is still scant [12].

## Conclusion

In sum, this report aims to increase physicians awareness about the various presentations of this rare disease and draw their attention to that when a patient presents with severe acute upper abdominal pain and fundamental imagings are in favor of acute gastric outlet obstruction, gastric volvulus can be an emergent diagnosis which requires immediate surgical consultation and intervention.
